# Supplementary material for: Green Health Partnerships in Scotland; Pathways for Social Prescribing and Physical Activity Referral
Source: Int J Environ Res Public Health. 2020 Sep 18;17(18):6832. doi: 10.3390/ijerph17186832 (PMC7560024; doi:10.3390/ijerph17186832)
Supplement: Supplementary file 1 [file ijerph-17-06832-s001.pdf]

Supplementary File

Green health interventions for social prescribing and physical activity referral; a qualitative study of the establishment of Green Health Partnerships in Scotland, UK

Sheona McHale [1,*], Alice Pearsons [1] , Lis Neubeck [1,2,] , Coral L Hanson [1]

[1] School of Health and Social Care, Edinburgh Napier University, Sighthill Campus, Edinburgh, EH11 4DN, UK; S.Mchale@napier.ac.uk, a.pearsons@napier.ac.uk, l.neubeck@napier.ac.uk, c.hanson@napier.ac.uk

[2] Sydney Nursing School, Charles Perkins Centre, John Hopkins Road, University of Sydney, Sydney, NSW 2006 Australia

* Correspondence: S.Mchale@napier.ac.uk

Table 1. Interview guides.

**GHP officers**	
**Question**	**Prompts**
Tell us a bit about the progress of the GHPs since you came into post	
What support have you had in developing the project?	What partnerships have been important in developing the project?<br>How does your role fit into the organisation that you are based in?<br>What support have you had from your line manager?<br>How this helped to support development of the project?
What has worked well?	Who has the project worked well for?<br>What circumstances have been important in influencing success?
What challenges have there been?	What has not worked so well?<br>Who has the project not worked so well for?<br>What partnerships have been difficult?<br>What circumstances have made it difficult to develop the project?
How do you think the GHPs have added value?	What new audiences have become involved in green exercise opportunities/nature-based interventions as a result of the project?<br>How has the GHP contributed to embedding prevention, early intervention and person-centred care into health and social care?<br>What gaps still need to be filled?

	How has the GHP improved opportunities for (and actual) volunteering?
How important has addressing health inequalities been in the project to date?	What steps have you taken to ensure that the GHP does not widen inequalities?<br>Who are you targeting?<br>Where are you targeting?<br>To what extent have the communities that you are targeting been involved in the design and delivery of the project?<br>How are you promoting the initiative?
What are the future challenges for the GHPs?	How do you think that the GHP's can be embedded in your area?<br>What organisations or people are key to this?<br>What might prevent future development?
Is there anything else about the GHPs that you would like to share with us before we finish?	

**GHP Steering Groups**	
**Question**	**Prompts**
Tell us about the progress of the GHP to date.	
What has worked well?	Who has the project worked well for?<br>What circumstances have been important in influencing success?
What challenges have there been?	What has not worked so well?<br>Who has the project not worked so well for?<br>What partnerships have been difficult?<br>What circumstances have made it difficult to develop the project?
How do you think the GHPs have added value?	What new audiences have become involved in green exercise opportunities/nature-based interventions as a result of the project?<br>How has the GHP contributed to embedding prevention, early intervention and person-centred care into health and social care?<br>What gaps still need to be filled?<br>How has the GHP improved opportunities for (and actual) volunteering?

What circumstances have prompted action for addressing health inequalities via the GHP in your area?	What steps have you taken to ensure that the GHP does not widen inequalities?<br>Who are you targeting?<br>Where are you targeting?<br>How are you promoting the initiative?
What are the future challenges for the GHPs?	What might prevent future development?<br>How can the GHP's be embedded in your area?<br>How will this work with existing structures?<br>What organisations or people are key to this?
Is there anything else about the GHPs that you would like to share with us before we finish?	

**Individual Interviews**	
**Question**	**Prompts**
Tell us what you know about the GHP project in your area.	
How does the GHP fit with the strategic direction of your organisation?	How can green health be positioned to play a role in the strategy of your organisation?<br>What progress has been made so far with this?<br>What challenges still exist in integrating green health to be a better strategic fit for your organisation?<br>How might these challenges be addressed?
How would you describe attitudes within the (health, social care and environmental sectors) in your area towards the use of green health activities in models of health promotion, recovery and care?	To what extent is the role of green health activities recognised?<br>What previous experience of cross-sector collaboration between environment and health existed in your area?<br>How have attitudes changed in recent years?<br>What still needs to be done?
Thinking about Scotland's six public health priorities and your own local priorities, where do you feel the contribution of the natural environment could be particularly significant?	How has or how could the GHP contribute to a sustainable, inclusive economy and reduce health inequalities?<br>How has or how could the GHP contribute to increased physical activity, better eating and healthy weight for the population?
How can the GHP contribute to addressing health inequalities in your area?	How could this benefit groups with heath conditions?<br>What is the importance of the natural environment in addressing mental health and wellbeing?

	What is the importance of the natural environment in addressing low levels of PA?<br>Which groups is this particularly important for?
What are the future challenges for the GHPs?	How positive do you feel about the future of the GHP approach (or, more generally, the contribution of green health)?<br>Based on your experience to date, how straightforward do you think it will be to upscale and mainstream this approach?<br>What organisations or people are key to this?
Is there anything else about the GHPs that you would like to share with us before we finish?	
